# Supplementary material for: Non-invasive super-resolution imaging through dynamic scattering media
Source: Nat Commun. 2021 May 25;12:3150. doi: 10.1038/s41467-021-23421-4 (PMC8149393; doi:10.1038/s41467-021-23421-4)
Supplement: Supplementary file 1 — Supplementary Information [file 41467_2021_23421_MOESM1_ESM.docx]

**Supplementary Information:**

**Non-invasive super-resolution imaging through dynamic scattering media**

Dong Wang^1,2,#^, Sujit K. Sahoo^1,3,#^ Xiangwen Zhu^1^, Giorgio Adamo^4^ and Cuong Dang^1,*^

^1^ Centre for Optoelectronics and Biophotonics (COEB), School of Electrical and Electronic Engineering, The Photonics Institute (TPI), Nanyang Technological University Singapore, 50 Nanyang Avenue, 639798, Singapore

^2^ Key Laboratory of Advanced Transducers and Intelligent Control System, Ministry of Education, and Shanxi Province, College of Physics and Optoelectronics, Taiyuan University of Technology, Taiyuan 030024, China

^3^ School of Electrical Sciences, Indian Institute of Technology Goa, Goa 403401, India

^4^ Centre for Disruptive Photonic Technologies, SPMS, TPI, Nanyang Technological University, Singapore 637371, Singapore

^#^ Dong Wang and Sujit K. Sahoo contribute equally.

* Corresponding author, E-mail: [HCDang@ntu.edu.sg](mailto:HCDang@ntu.edu.sg)

## Optical experiment setup

The optical setup for our experimental demonstration of stochastic optical scattering localization imaging (SOSLI) is depicted schematically in Supplementary Fig. 1. It consists of two parts: the object simulator and the imaging setup. The former is designed for convenient generation of various objects with blinking point sources. To create microscopic objects, we certainly do not want the projector's projection lens as it magnifies images to a huge screen. We replace the projection lens of a commercial projector (Acer X113PH) by a microscope objective (40x, numerical aperture: NA=0.65, not an infinity-corrected objective) to de-magnify projector pixels to squares of $1.34 \times1.34$ µm^2^ at the object plane. There is an iris placed at the projected plane of the projector to remove its stray light. And the second iris is at the object plane to further block unwanted light from the projector and environment. Light from the object passing through both scattering media and the imaging iris is captured by a camera sensor (Andor Neo 5.5, 2560×2160 pixels, and 6.5-µm pixel size). The scattering media are a ground glass diffuser (a static one) or a fresh chicken eggshell membrane (a dynamic one) in our demonstration. An optical filter (Thorlabs FB550-10, 550 nm wavelength, and 10 nm full-width at half-maximum) is mounted on the camera to narrow the optical spectrum. Blinking point sources are generated by randomly blinking projector pixels. For invasive measurement of the point spread function (PSF), only one center pixel is turned on. The exposure time to capture each speckle pattern is 5-10 seconds to gain good signals.


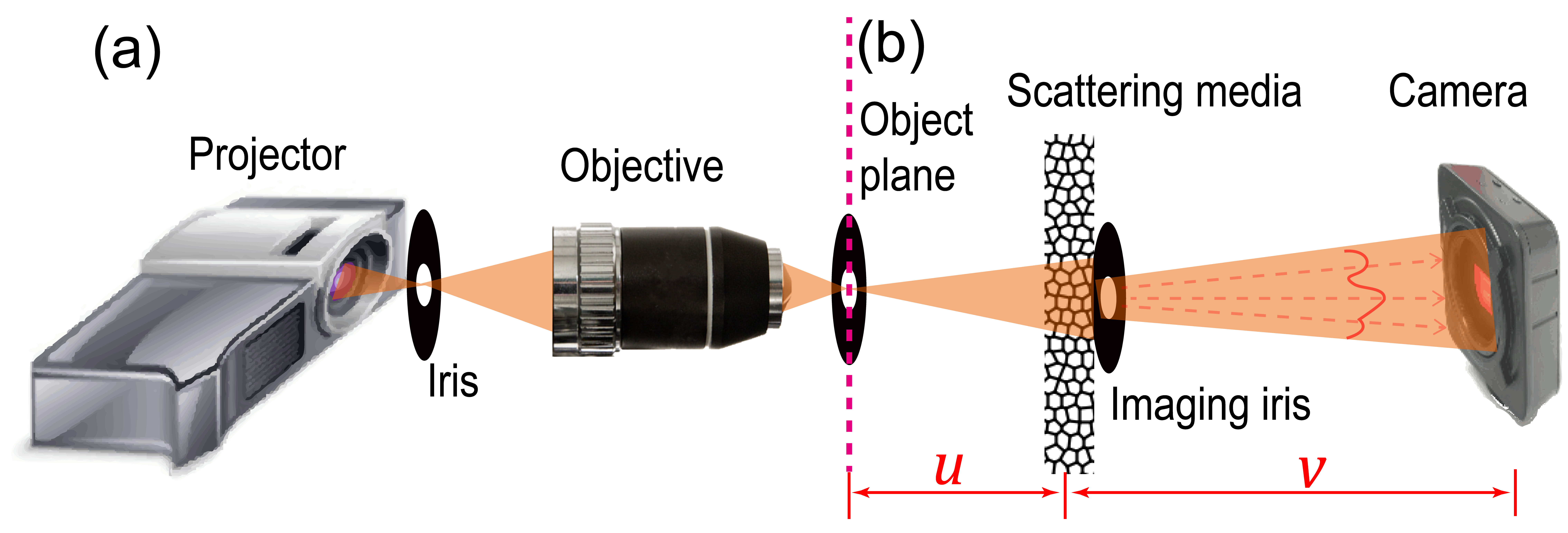


**Supplementary Fig. 1:** **Optical setup to demonstrate SOSLI for non-invasive super-resolution imaging through strongly scattering media.** **(a)** Object-simulator, which is designed for generating various microscopic objects at the object plane. **(b)** Simple optical configuration for imaging setup where *u* = 10 mm and *v* = 100 mm.

Although the projector’s projection lens is removed so that it does not project to a huge screen, there are still multiple unknown optical components inside for various projector functions such as zooming, keystone correction, etc. In fact, the projector still projects images to the plane of the first iris after projection lens removal. Hence, the projector's remaining optics plays as a relay lens from the digital micromirror device (DMD) to the first iris plane. The objective lens is placed just 5 centimeters from the first iris plane to de-magnify the image further. We simply use the objective lens as a short-focal-length lens rather than set it up to achieve 40X de-magnification. One can do the same with a normal short-focal-length lens. We would like to note that there is also a diffraction limit for the projection image; and more de-magnification requires further distance from the objective to the first iris plane, leading to reduced signal.

Due to the unknown remaining relay optics in the projector, we cannot calculate the demagnification factor from the DMD to the object plane. We measure the pixel size of 1.34 µm by a mechanical approach. We turn on 200 pixels to make a few-hundred-micrometer bright line. A blade is then moved along the line to block a portion of it and the transmitted light intensity is monitored by a power meter. We used a differential actuator to control the blade position with sub-micrometer precision. By coordinating the transmission intensity with the blade position, the line length and then the pixel size can be calculated.

The optical configuration for the imaging side is relatively simple. To get easy success, we choose the distances *u* and *v* to achieve adequate magnification while maintaining acceptable signal and sufficient memory effect. We first select the smallest *u* so that our phase retrieval algorithm is still successful; this confirms our sample is within the memory effect of the scattering media. Then the distance *v* is then chosen so that the magnification (*M = v/u*) is significantly large to resolve objects at high resolution by camera pixels. In non-invasive applications, neither *u* nor the memory effect region is our choice, but both determine the maximum size of measurable objects. Therefore, we simply need to put the camera and adjust the camera position to capture the signal for SOSLI. A smaller *v* will gain the SNR at the cost of reduced magnification and vice versa.

## The current state-of-the-art non-invasive and invasive imaging

We conduct experiments to demonstrate the current state-of-the-art non-invasive and invasive imaging through a 120-grit ground glass diffuser using the experimental setup shown in Supplementary Fig. 1. A non-invasive image is retrieved from the autocorrelation of a single-shot speckle pattern by applying the phase retrieval algorithm. An invasive image is the deconvolution of a single-shot speckle pattern with an invasively measured PSF. The imaging iris diameter is set as 1 mm, 2 mm and 3 mm that correspond to NAs of 0.05, 0.1 and 0.15, respectively, and diffraction limits of 6.7 µm, 3.4 µm and 2.3 µm respectively. We can easily see the effects of NA on the resolution from the results given in Supplementary Fig. 2. An imaging system is a low pass filter, where higher NA (higher cut-off frequency) provides higher resolution (i.e. sharper) images than lower NA does. Because of the adverse effects from noise, the camera’s dynamic range and dark counts on the phase retrieval algorithm’s performance, the single-shot non-invasive images have a slightly lower resolution than the diffraction limit. If these effects are too high, the algorithm may not even converge. On the other hand, the deconvolution images have a slightly higher resolution than the diffraction limit because deconvolution recovers and enhances the image’s high-frequency components.


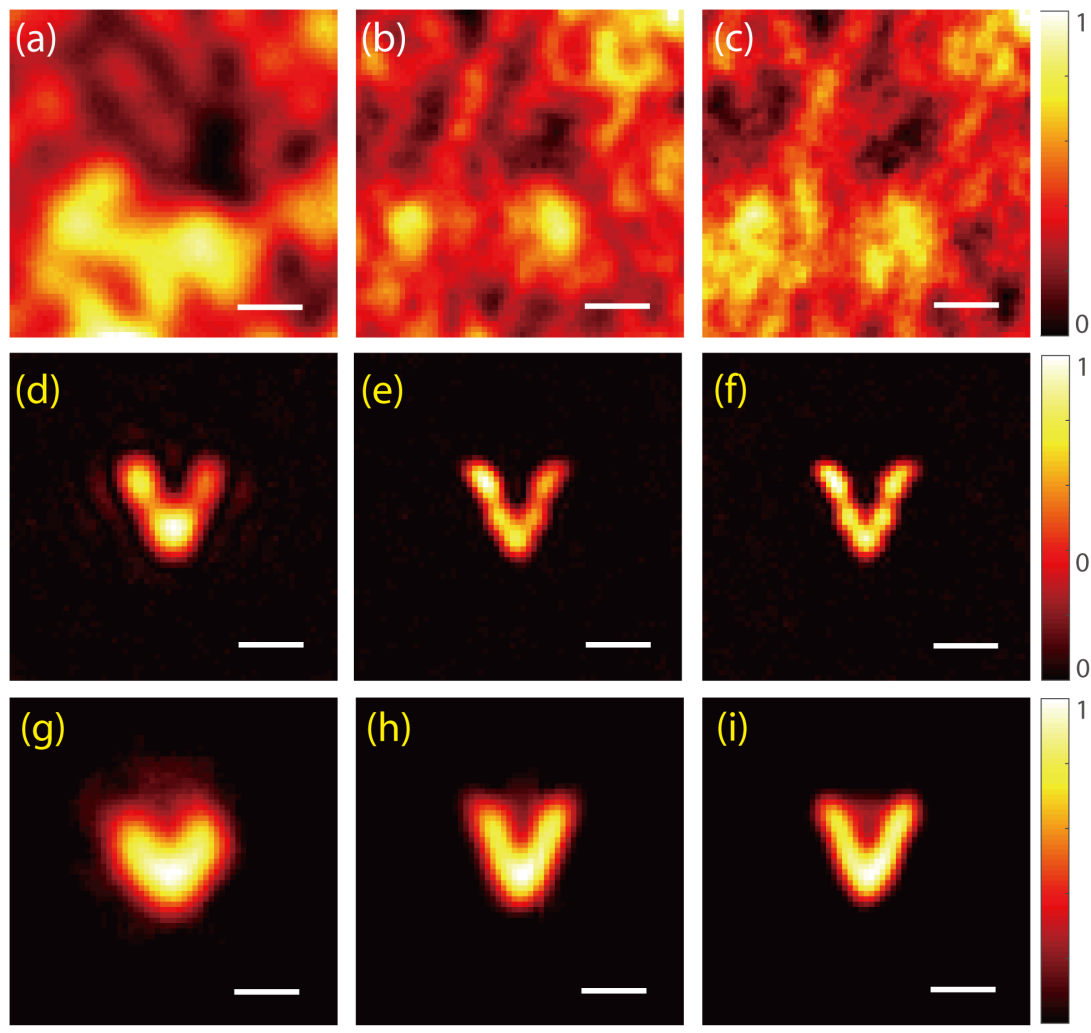


**Supplementary Fig. 2: The effect of NA on the resolution for invasive and non-invasive imaging through scattering media.** **(a-c)** The speckle patterns of the same object with NAs of 0.05, 0.1, and 0.15, respectively. Experimental results of the state-of-the-art invasive **(d-f)** and non-invasive **(g-i)** imaging through the 120-grit ground glass diffuser with the speckle patterns in (a-c) respectively. Scale bar: 10 camera pixels, equivalent to 6.5 µm on the object plane.

## Multiple estimated PSFs from a single stochastic speckle pattern

From a series of stochastic speckle patterns recorded for SOSLI, we pick up one pattern with high contrast (Supplementary Fig. 3a). The iterative phase retrieval algorithm is utilized to retrieve the point source frame at low resolution as presented in Supplementary Fig. 3b-c. Two similar frames shifted from each other can be retrieved from a single speckle pattern by two different runs of the algorithm because autocorrelation only keeps the relative point source positions, while losing their exact positions. Supplementary Fig. 3d-e present the point source positions after localization. The localized-source images show very clean point sources, removing all the noise or artifacts of the phase retrieval algorithm. Supplementary Fig. 3f-g show the estimated PSFs calculated from a single speckle pattern (Supplementary Fig. 3a) and two different phase retrieval/localization results (Supplementary Fig. 3d-e). Supplementary Fig. 3i-j present the point source positions localized after deconvolution of another speckle pattern (Supplementary Fig. 3h) with two estimated PSFs in Supplementary Fig. 3f-g. The point source positions in Supplementary Fig. 3i-j automatically align very well with those in Supplementary Fig. 3d-e, respectively because they are reconstructed from the same estimated PSFs. The absolute positions of point sources and PSF are not important in our SOSLI. They do not affect our results, and usual imaging techniques are not concerned about absolute position. But we emphasize that the relative positions of the point sources are required for image formation.


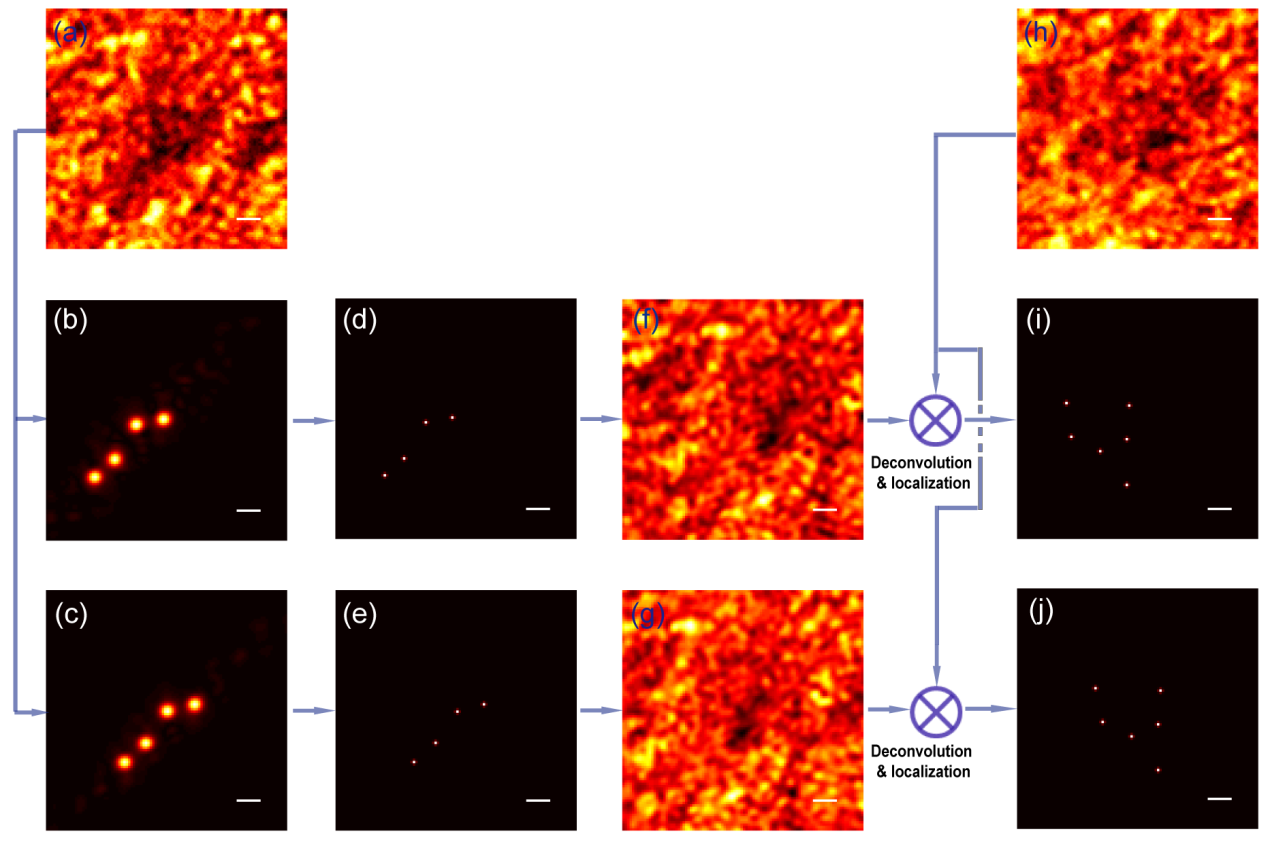


**Supplementary Fig. 3:** **Deconvolution process with different estimated PSF. (a)** A typical stochastic speckle pattern. **(b-c)** Typical images retrieved from autocorrelation by the phase retrieval algorithm. **(d-e)** Localized-source images. **(f-g)** Estimated PSFs. **(h)** Another stochastic speckle pattern. **(i-j)** Point source positions calculated from a single speckle pattern and two estimated PSFs. Scale bar: 10 camera pixels, equivalent to 6.5 µm on the object plane.

## SOSLI with PSFs estimated from different speckle patterns

During our implementation of SOSLI, we randomly choose a high-contrast pattern out of multiple collected speckle patterns for estimation of the PSF. Interestingly, different speckle patterns give slightly different estimated PSFs. The differences are not only an arbitrary shift from each other but also the speckles themselves (Supplementary Fig. 4a-f and 5b-c). However, the reconstructed images from these estimated PSFs are very similar (Supplementary Fig. 4g-i) with different shifts. It implies that we only estimate the main features of the PSF, which is sufficient for reconstruction. We test the similarity of these three estimated PSFs by calculating the correlation among them. The autocorrelation of PSF1 shows its random speckle nature with a bright spot (Gaussian profile) at the center (Supplementary Fig. 5a). The correlation of PSF1 with the other PSFs indicates an off-center bright spot with some background (Supplementary Fig. 5b-c), implying that these PSFs share the main features and shift from each other. The clear, bright spot allows its center to be located then the relative shift between PSFs to be obtained. The relative shifts (Supplementary Fig. 5d) are precisely equal to the relative shifts between the retrieved objects (Supplementary Fig. 4g-i).


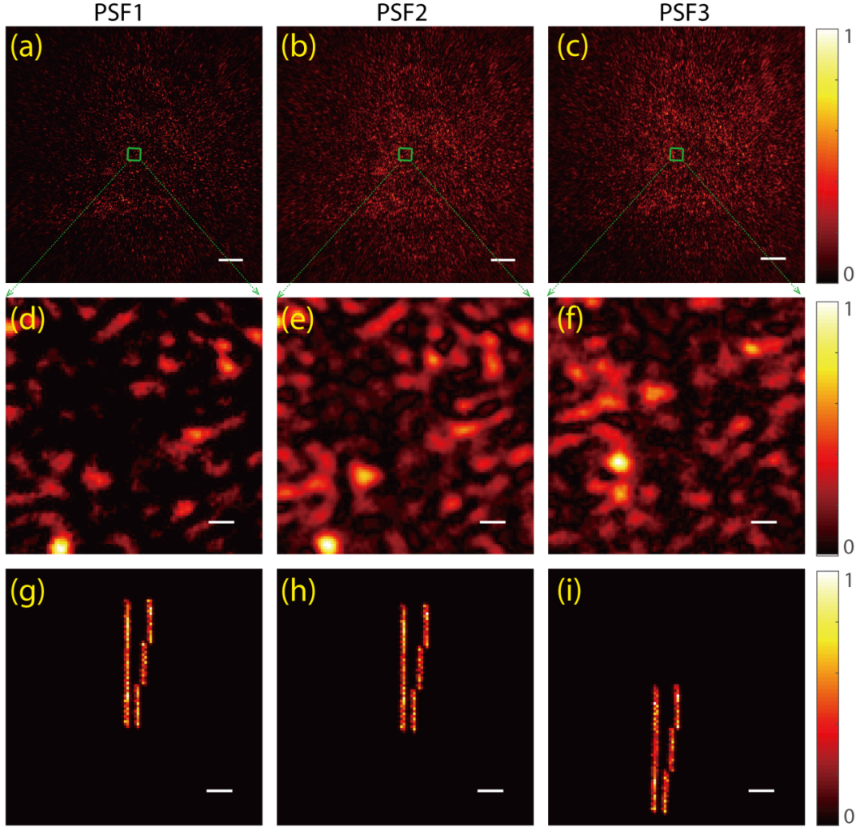


**Supplementary Fig. 4: Retrieved results of an object with the PSFs estimated from different speckle patterns arbitrarily chosen.** **(a-c)** The estimated PSFs (full scale), **(d-f)** The zoomed-in center part of corresponding PSFs in a-c. **(g-i)** The corresponding super-resolution SOSLI results. Scale bar: 200 camera pixels for a-c, and ten camera pixels for d-i, equivalent to 6.5 µm on the object plane.

The observation is interesting, meaningful and useful. Because of the localization, we can easily remove the background artifact and noise in the phase retrieval images and deconvolution images. Therefore, the SOSLI approach can tolerate more error in PSF estimation. This not only explains how and why the SOSLI should work very well in static scattering media, but also inspires us to conceive a successful solution for dynamic scattering media in section 8.


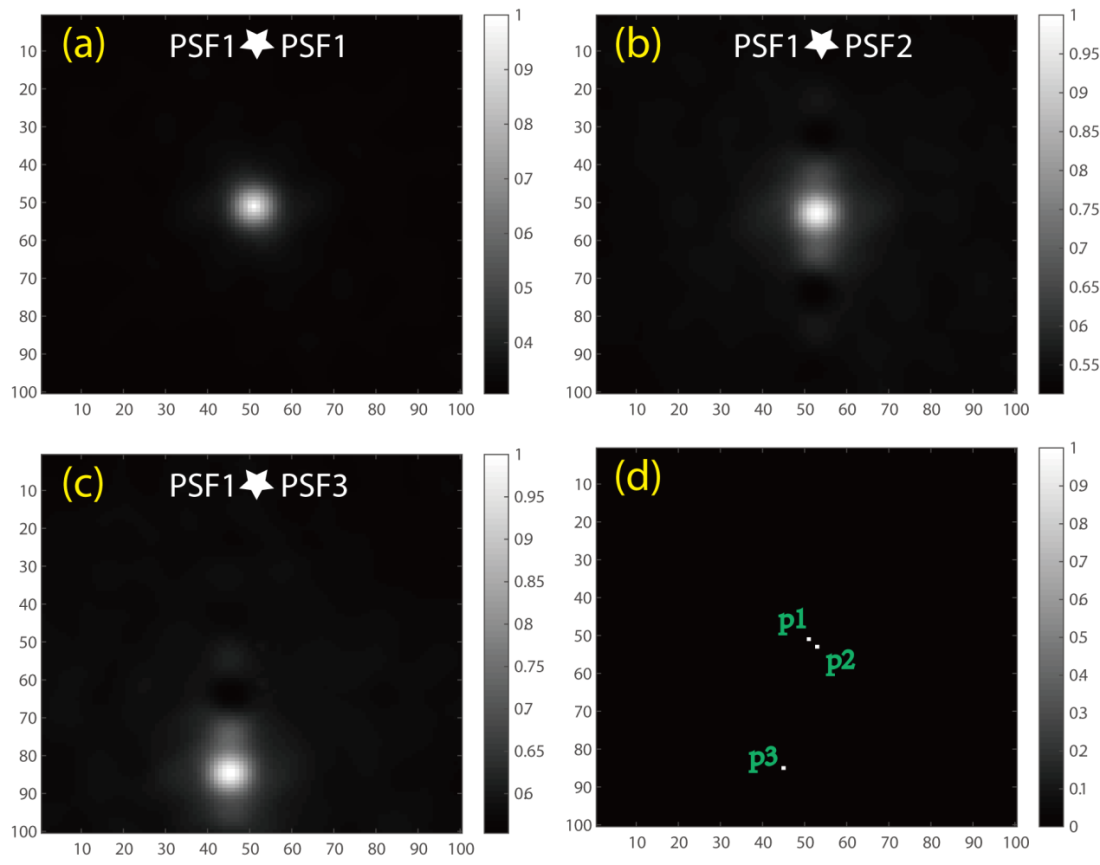


**Supplementary Fig. 5: Cross-correlations of the PSF1 with the other PSFs to obtain the relative shifts between them.** **(a-c)** The correlation patterns. **(d)** The relative shifts between PSF1 with other PSFs. The numbers on the edges of the image indicate the pixel numbers.

## SOSLI with different numbers of stochastic patterns.

In our experiment, the object to be imaged through a diffuser constitutes multiple blinking point sources. The SOSLI technique reconstructs a super-resolution image from multiple stochastic patterns, and the quality of the reconstructed image will increase with the number of frames. We characterize the reconstructed images with various numbers (n=200, 400, 800, and 8000) of the randomly blinking point source patterns which are used Fig. 3c (main text). The results are shown in Supplementary Fig. 6, where Supplementary Fig. 6e-h show the results of Supplementary Fig. 6a-d after bicubic interpolation processing. It is obvious that reconstruction with 8000 stochastic patterns gives the best image quality (Supplementary Fig. 6d&h). However, 300-400 frames are sufficient to reconstruct our simple object (Supplementary Fig. 6b&f).


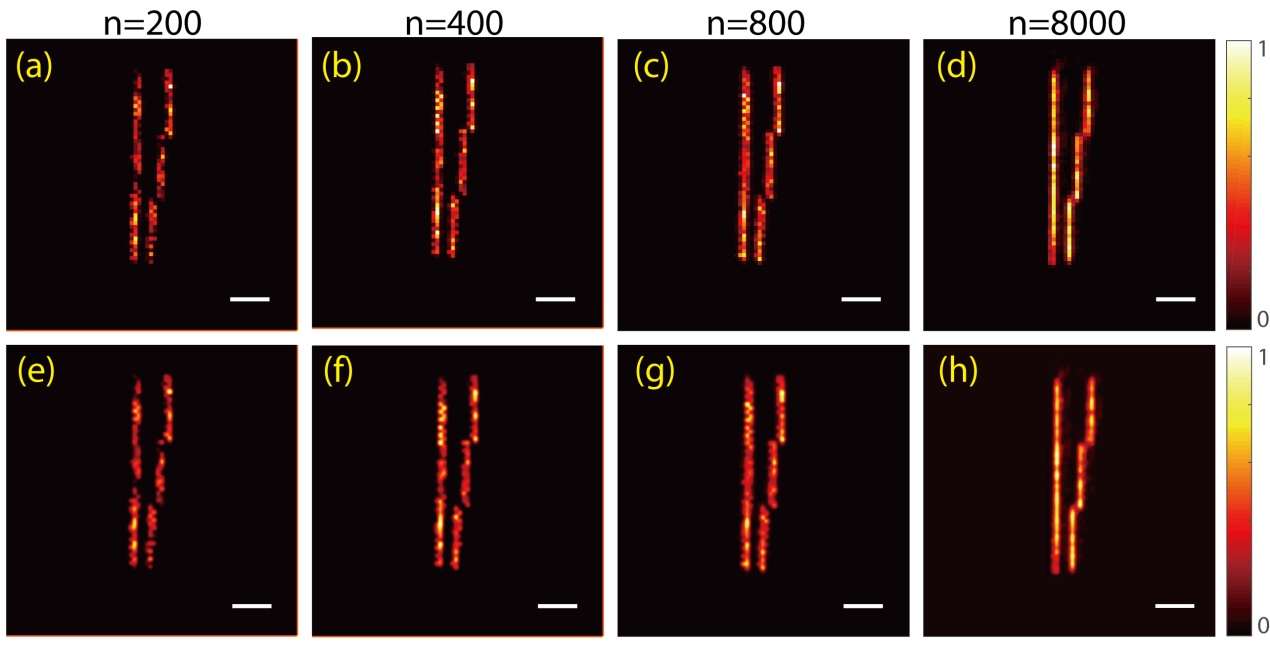


**Supplementary Fig. 6: Retrieved results of an object by SOSLI with the different numbers of stochastic patterns**. **(a-d)** The raw results from SOSLI. **(e-h)** The results after bicubic interpolation processing that makes images smoother. Scale bars: 6.5 µm.

## Deconvolution results with a decorrelated PSF


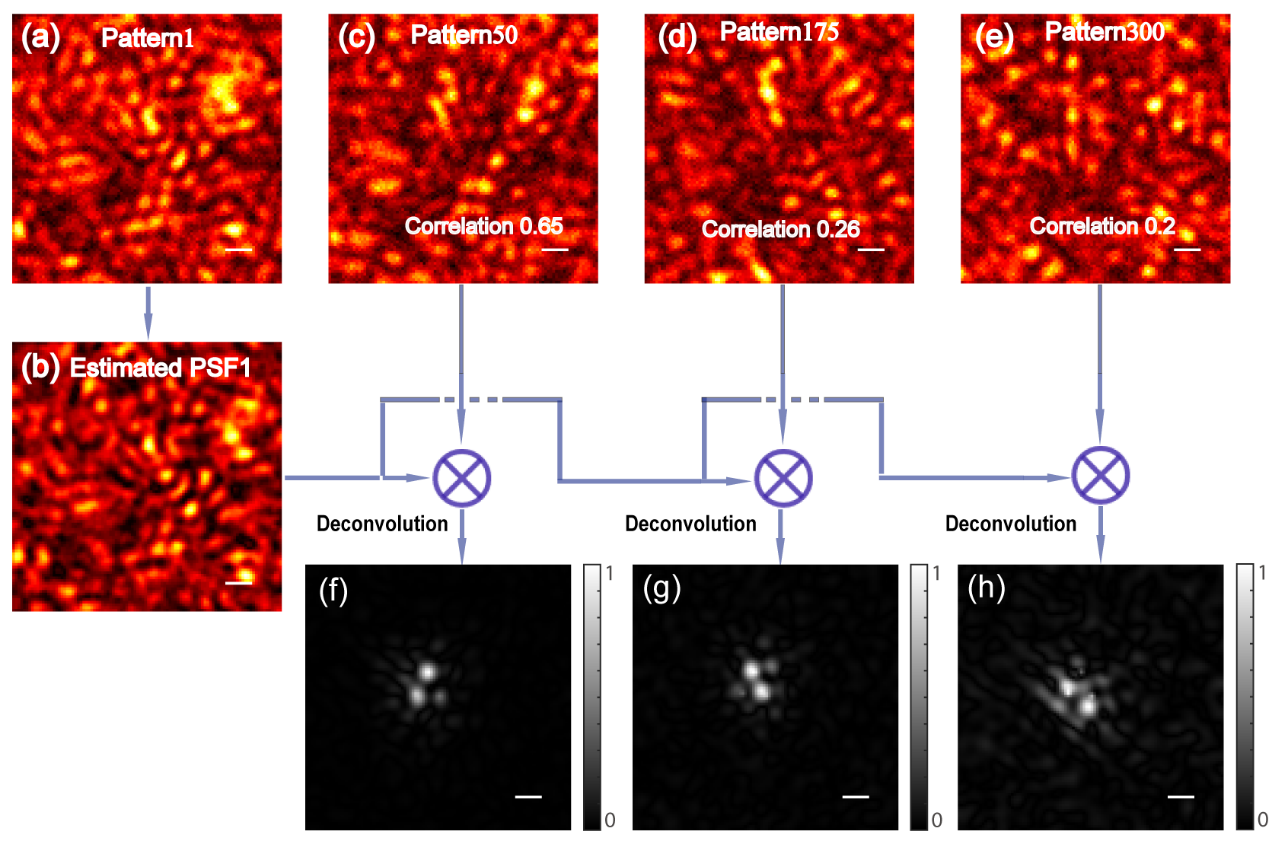


**Supplementary Fig. 7: Deconvolution results with a decorrelated PSF of the chicken eggshell membrane. a)** The first stochastic speckle pattern is chosen to estimate the PSF. **b)** The estimated PSF from the first stochastic speckle pattern (PSF1). **c-e)** The stochastic speckle patterns with the membrane’s decorrelation (the correlation coefficients for the pattern number are presented in the main text Fig. 5a: 0.65, 0.36, 0.2 respectively). **f-h)** The deconvolution results of the speckle patterns in c-e, respectively, with the estimated PSF1 from the first speckle pattern. There are increasing artifacts in deconvolution images with an increase of scattering media’s decorrelation. Scale bar: 10 camera pixels, equivalent to 6.5 µm on the object plane.

## Adaptive SOSLI for dynamic scattering media


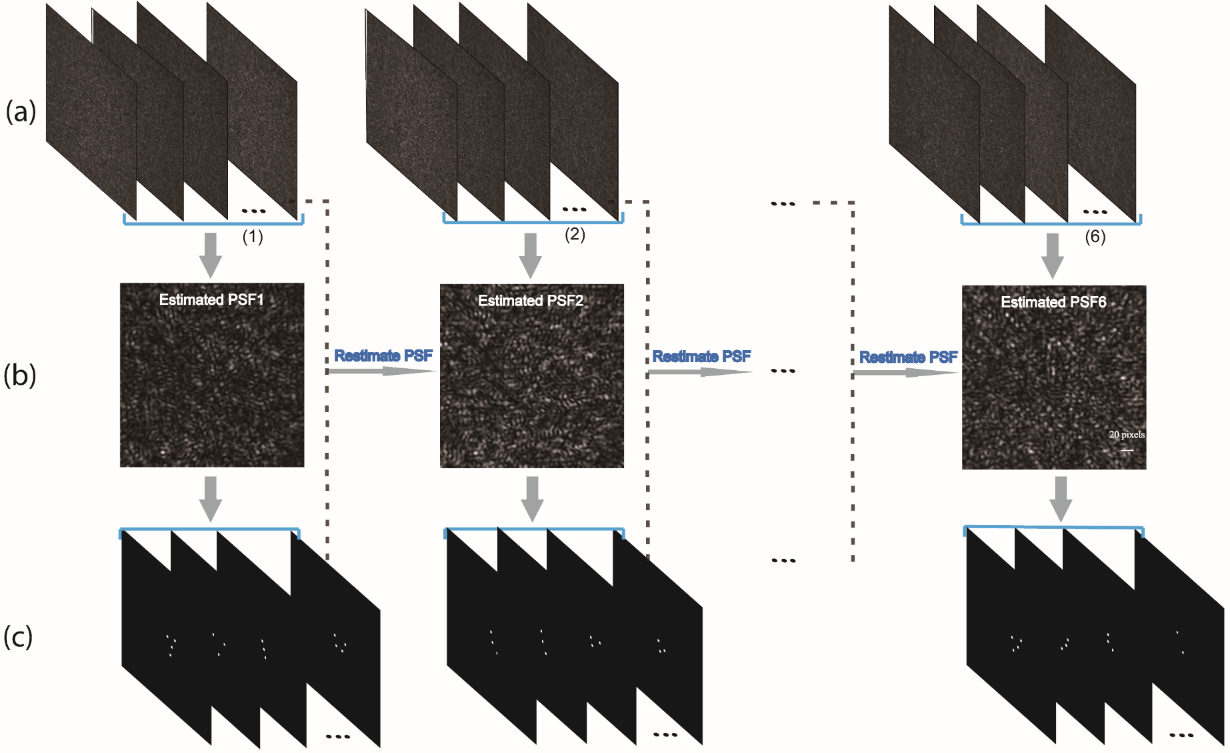


**Supplementary Fig. 8: Schematic diagram of the adaptive SOSLI for super-resolution imaging through dynamic scattering media. (a)** Multiple groups of speckle patterns. **(b)** Strategy to estimate adaptive PSFs. **(c)** Localized point sources in each speckle pattern.

For dynamic scattering media, a series of speckle patterns are captured with slightly different scattering characteristics. The proposed adaptive SOSLI divides all these speckle patterns into multiple groups (Supplementary Fig. 8a). The number of stochastic patterns for each group depends on the decorrelation time and image acquisition time. The requirement is that every two adjacent groups have some finite correlation. We do SOSLI with the first group and achieve estimated PSF1 (Supplementary Fig. 8b) and multiple frames of localized point sources (Supplementary Fig. 8c). For finite-dynamic scattering media, the decorrelation within a small group of images has not yet affected SOSLI performance. We then use the last speckle pattern and its localized point sources to re-estimate the PSF, which is PSF2 (Supplementary Fig. 8b). The PSF2 is utilized for deconvolution then localization for all the speckle patterns in the second group (Supplementary Fig. 8c). The process is continued for all the groups, and a full set of localized-source frames is achieved (Supplementary Fig. 8c) to reconstruct a super-resolution image by superposition.

## PSF alignment for highly dynamic scattering media

The most adaptive SOSLI approach utilizes the phase retrieval algorithm to correct the deconvolution image (with the PSF estimated from previous speckle pattern), while the deconvolution image is used to maintain no-shifting and no-flipping between two retrieved images for superposition. An additional step that checks the shifting and flipping condition of the estimated PSF with the previous one could allow us to reconstruct super-resolution images more reliably, thus enhancing resolution.


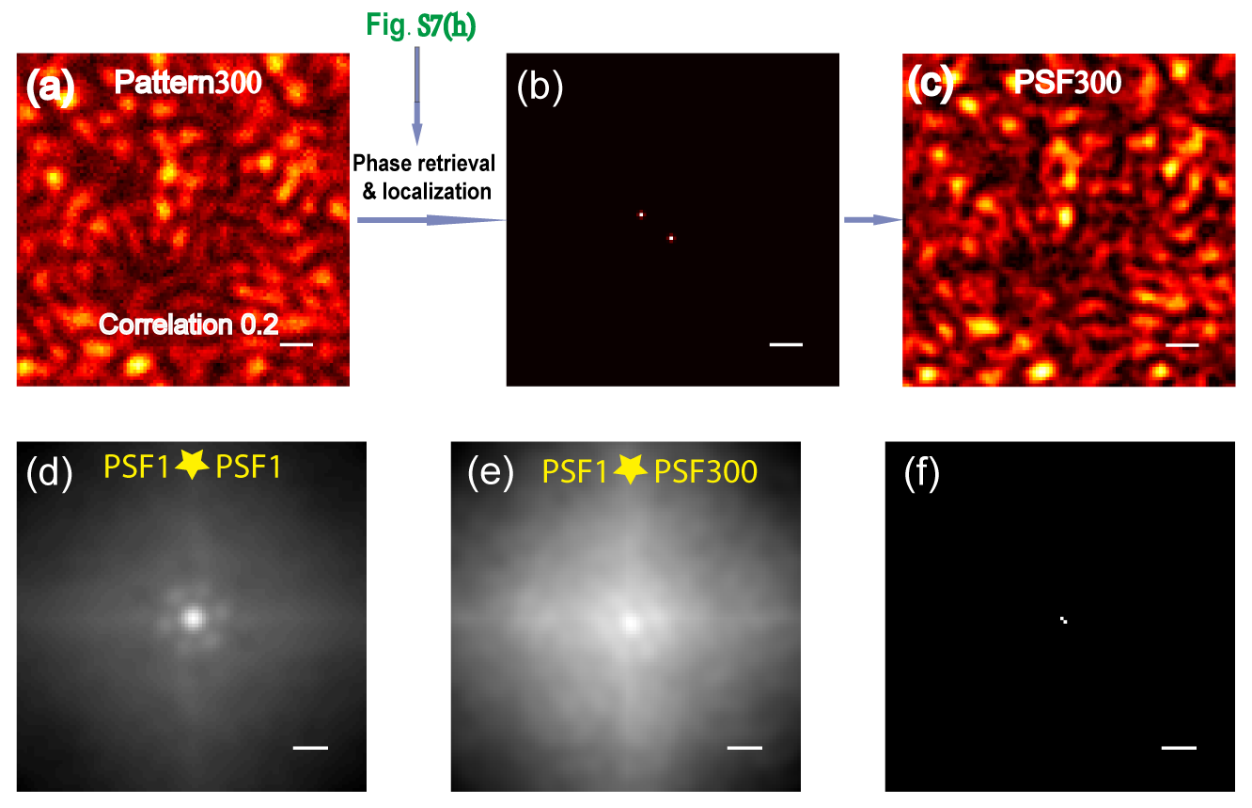


**Supplementary Fig. 9: The most adaptive approach for SOSLI to mitigate highly dynamic scattering media. a)** A speckle pattern (pattern 300) taken when scattering media is largely decorrelated (the correlation is only 0.2). **b)** A point source frame recovered from the speckle pattern in (a) by the phase retrieval algorithm with the initial guess from Supplementary Fig. 7(h) and then by localization. **c)** The estimated PSF from the speckle pattern in (a) and the point source frame in (b). **d)** The autocorrelation of PSF1, which is Supplementary Fig. 6(b). **e)** The correlation between PSF1 and PSF300 (in figure c). **f)** The centers of brightest spots in d&e, showing the relative shift between two PSFs, which is also the relative shift between two point source frames derived from speckle pattern #1 and #300 by phase retrieval and localization. Scale bar: 10 camera pixels, equivalent to 6.5 µm on the object plane.

Here, we take two speckle patterns #1 and #300 as an example for two consecutive shots of highly dynamic scattering media, where the correlation is expected to be only 0.2. Supplementary Fig. 7h presents the deconvolution image of speckle pattern 300 with the PSF1, which is estimated from speckle pattern 1. We use Supplementary Fig. 7h as the initial guess in phase retrieval algorithm for speckle pattern 300 (Supplementary Fig. 9a) to achieve a high-quality image. Then, the localization process provides precise point source positions (Supplementary Fig. 9b) with respect to those from pattern 1 for superposing in SOSLI. In some rare cases, noise and large decorrelation of scattering media in these two shots might cause some errors in relative positions between two reconstructed point source frames, degrading the resolution of SOSLI. To confirm and realign the relative position of reconstructed point source frames, we re-estimate the new PSF as PSF300 (Supplementary Fig. 9c), then calculate the correlation of PSF1 with PSF300 as presented in Supplementary Fig. 9e which has a bright spot on a strong background. The center of the bright spot can be identified by the localization algorithm. The offset of this correlation center from the image center (Supplementary Fig. 9f) is the relative shift between two PSFs, which is also the relative shift between two reconstructed point source frames. If we fine-tune the correction rate in the phase retrieval algorithm carefully, we can achieve no offset most of the time, i.e. the cross-correlation pattern between PSF1 and PSF300 is peaked at the center (similar to the autocorrelation of PSF1 in Supplementary Fig. 9d) with, certainly, a lot more background noise. However, such fine-tuning of the phase retrieval algorithm parameters depends on the context and nature of the images. Therefore, we should always calculate the relative shift between 2 phase retrieved images, then realign them before superposing to achieve better SOSLI results.

## SOSLI with low-photon point sources

SOSLI relies on speckle patterns which are formed by photons from point sources. For each pixel in a speckle pattern, the intensity represents the probability that photons land at that pixel (Supplementary Fig. 10a). If the number of photons is insufficient, the speckle pattern is “underdeveloped”, i.e. there is uncertainty in the captured speckle patterns (Supplementary Fig. 10b-c). The first step in SOSLI is to perform a low-resolution image recovery for any pattern of the blinking point sources using the phase retrieval algorithm and localization. Therefore, the success of SOSLI relies on the success of this first step. Hybrid-Input-Output (HIO) Fienup algorithm is used in our phase retrieval step, which is widely used for its simplicity and speed. However, its success varies, depending on the initial guess. Therefore, the usual practice is to run the algorithm with a few random starting guesses and choose a good result. In general, this algorithm converges for fully developed speckle patterns with almost any initial guess. An underdeveloped speckle pattern due to the low photon budget would corrupt the signal, thus, posing a big challenge for this step. Here, we perform a simulation to check the success rate at various photon budget as detailed below.

A typical noise-free speckle pattern is used as the “fully-developed” speckle pattern (Supplementary Fig. 10a). The speckle patterns are then quantized to 32-bit numerical precision, which can be treated as a 2^32^-photon counting capacity of each pixel. Taking this fully developed quantized speckle pattern as the discrete photon distribution model (i.e. the probability mass function), samples of scattering photons are generated by Monte Carlo simulation. The image size is taken as 1024x1024 pixels, and the photon budget is quantified as average photon per pixel (PPP). Therefore, the total number of photons used for various speckle patterns is $PPP\times1024\times1024$, which is distributed on 1024x1024 pixels according to the probability. Supplementary Fig. 10b-c show two typical speckle patterns with PPP = 0.7. The uncertainty of the speckle pattern can be easily observed. For our simulation, a total of 60 random realizations are obtained for each PPP scenario; and for each realization of the speckle pattern, the phase retrieval algorithm is deployed with 60 random starting guesses. There are 3600 trials for each PPP.


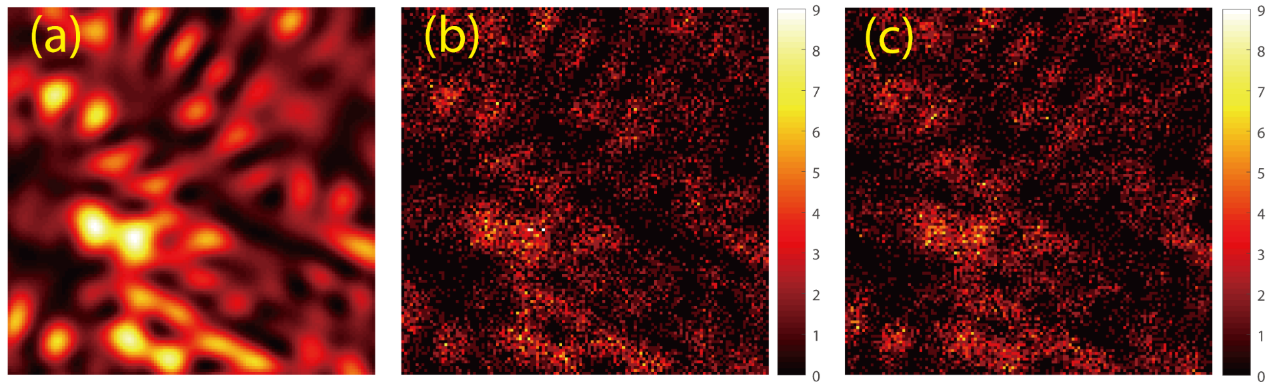


**Supplementary Fig. 10:** **(a)** A zoomed-in portion (128x128 pixel) of a fully developed speckle pattern from one stochastic point source pattern. **(b-c)** typical “underdeveloped” speckle patterns with the same PPP = 0.7.

The success rate is decided from the successful recovery of the object out of these 3600 trials. Here, a successful recovery is defined as the exact relative positions of the blinking point sources after the phase retrieval and localization. The sparsity of the object also helps phase retrieval algorithm to converge better. We choose the sparsest point source pattern with only 2 point sources to examine the lowest photon budget requirement. For an accurate classification of success, we compare the vector connecting two ground-truth point sources with the vector connecting two localized point sources (as well as its central flipping versions). The matching of vectors at the single-pixel level implies the success of this step without worrying about absolute position. Supplementary Fig. 11a-c describe one successful instance corresponding to PPP = 1, the phase-retrieved image (Supplementary Fig. 11a) is good enough to localize point sources correctly (Supplementary Fig. 11b) compared to the ground truth (Supplementary Fig. 11c). Supplementary Fig. 11d plots the success rate as a function of PPP. With 1 photon per pixel, the chance of success in phase retrieval and localization is about 11%. This probability drops to 2.8% with PPP = 0.8.


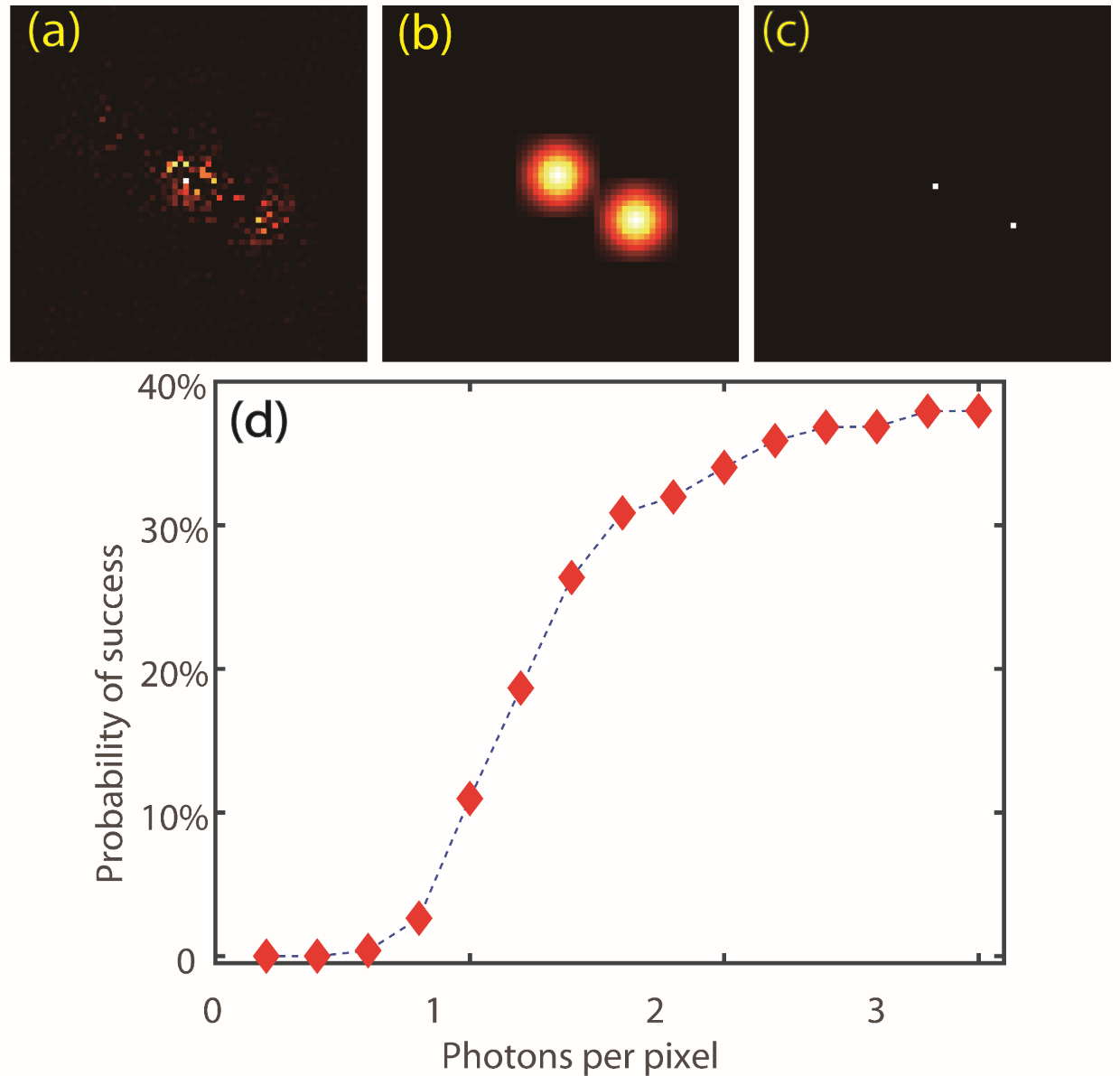


**Supplementary Fig. 11:** **(a)** A recovered object from a speckle pattern (PPP = 1.0) using phase retrieval. **(b)** Localization algorithm successfully finds 2 point sources from (a) using a 5x5 gaussian window, then the centers of these gaussian hot spots are identified. **(c)** The actual object. **(****d)** The success rate of phase retrieval and localization is stated as the probability of success in a trial for various photons per pixel scenario.

After successfully recovering one stochastic object using the phase retrieval algorithm and localization, the next step is to estimate the PSF using the recovered object and the low photon count image. Then the estimated PSF is used to recover the remaining stochastic point sources from their low photon count speckle patterns. Therefore, in the next simulation, we will evaluate the success rate in recovering a different stochastic point source pattern from the successful estimated PSF. Since the estimated PSF is different for different random realizations of the speckle image, we take 60 estimated PSFs from the previous successful step at each PPP. Using each estimated PSF, a different stochastic source pattern is recovered by deconvolution followed by localization from 60 random realizations. Overall, we have 3600 trials to examine the success rate of deconvolution and localization process. Supplementary Fig. 11a-c show one example of estimated PSF with PPP = 0.7, the deconvolution image, and the localized point sources, respectively. Supplementary Fig. 11d plots the success rate evaluation at various PPP values. It shows that the deconvolution process is robust to low photon count in the evaluated range. It is also understandable that phase-retrieval is the bottleneck of SOSLI; if the signal is good enough for the phase retrieval algorithm, the deconvolution will be successful.


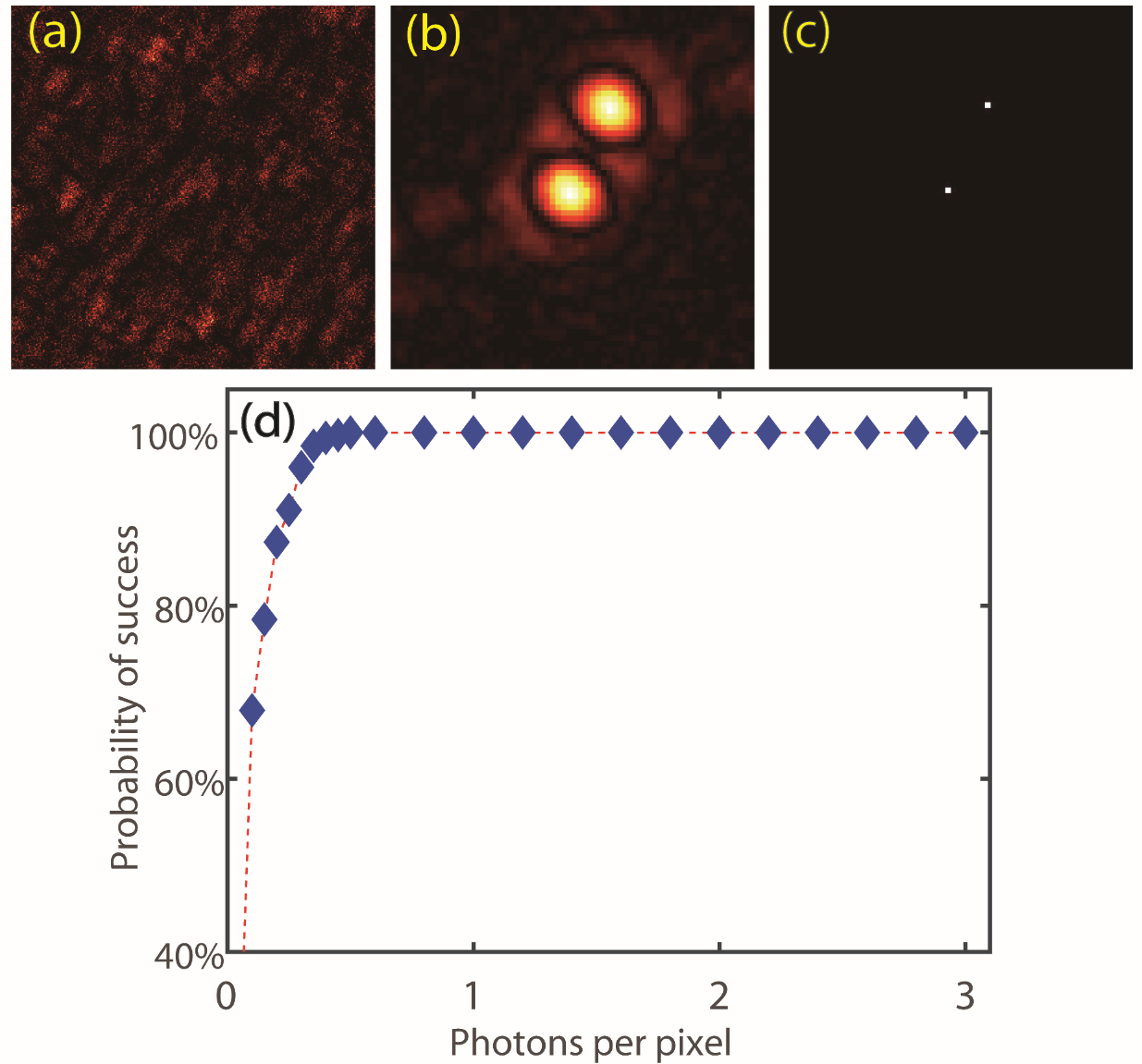


**Supplementary Fig. 12:** **(a)** An estimated PSF using the successful estimated object from the phase retrieval step. **(b)** A reconstructed stochastic object by deconvolution using the estimated PSF. **(c)** A super-resolution image after localization. **(d)** The success rate of deconvolution and localization is stated as the probability of success in a trial for various photons per pixel scenario.

## Mean free path of scattering media.

To understand the scattering power of scattering media, we measure the mean free path (MFP) of various 3D scattering media (including eggshell membrane) by following the method presented in by Amaury Badon et al.^1^ The experimental setups for both scattering MFP and transport MFP are illustrated in Supplementary Fig. 13.


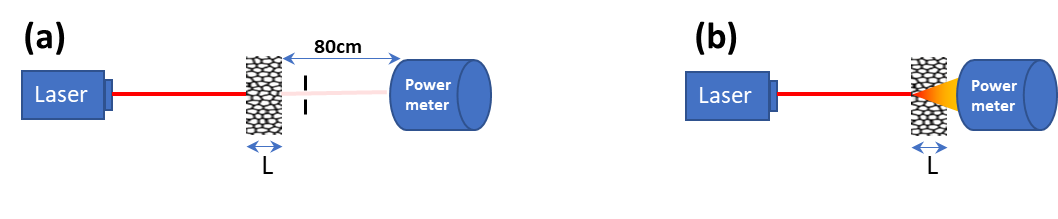


**Supplementary Fig. 13:** Experimental setups to measure the scattering MFP (a) and transport MFP (b) of various scattering media. The laser wavelength is 680nm. **(a)** Power meter at the far distance and an iris with 2 mm diameter are used to measure only ballistic light intensity *I_b_*. **(b)** The power meter is very close to the scattering media to collect most of the transmitted diffusion light.

The ballistic light intensity (*I_b_*) is a function of the media thickness (*L*) and its scattering MFP (*l_s_*)

$I_{b}=I\exp(-L/l{}_{s})$ (1)

The experiments were done with different numbers of scattering layers to estimate the ratio *L/l_s_* and understand the scattering strength in the media. Supplementary Fig. 14 below presents the experimental results for super thin wax papers (a), chicken eggshell membranes (b) and printing papers (c).


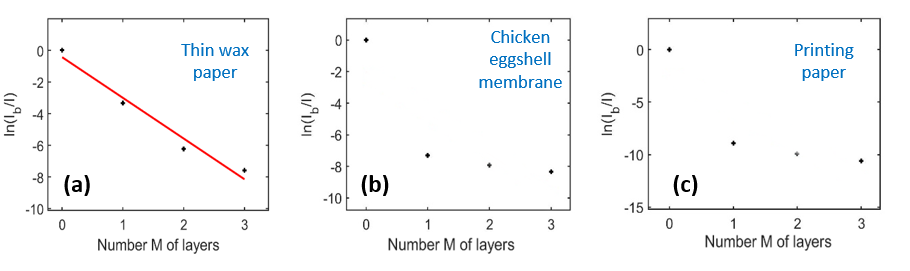


**Supplementary Fig. 14:** Ballistic photon intensity as a function of scattering layer number for three media: (a) thin wax papers, (b) chicken eggshell membranes and (c) printing papers

For thin wax papers, the ballistic light intensity follows the equation (1) nicely. We can estimate the thickness, *L*, of each paper sheet is about 3*l_s_*.

For chicken eggshell membranes and printing papers, we see an immediate drop of intensity with just a single layer. Then the intensity decreases slowly when adding the second and third layer. This implies that the thickness of a single layer is already significantly larger than the scattering MFP. If we simply take the first two data points (i.e. no scattering media and single layer scattering media) to fit the equation (1), we can estimate the lower limit of the scattering strength. The results show very strong scattering effect in both chicken eggshell membranes and printing papers with the *L/l_s_* ratio is “at least” 7.3 and 9, respectively.

For chicken eggshell membranes and printing papers: L>>l_s_; therefore, most of the transmitted light is scattered several times while propagating through a single layer, there is no ballistic photon. In this case, the diffusion approximation becomes valid, where the transport MFP (*l_t_*) is the characteristic of the diffusion model. The total transmission coefficient can be calculated as follows.

$T=\frac{z_{0}+l_{t}}{L+2z_{0}}$ (2)

where $z_{0}=\frac{2}{3}l_{t}\frac{1+R}{1-R}$, with R is the internal reflection coefficient.

According to the reference^1^, mean refractive index for paper is 1.5, R = 0.57 and therefore *z_0_ =* 2.4*l_t_*. We also use the same parameters for the chicken eggshell membrane. The experimental data are plotted in Supplementary Fig. 15 for two diffusion cases. From the slope of the linear fitting line, we can estimate the ratio *L/l_t_* for a printing paper sheet and a chicken eggshell membrane layer as 5.6±0.8 and 3.6±0.5, respectively. The thickness *L* of both a printing paper sheet^1^ and a chicken eggshell membrane^2^ is about 90 µm; we can calculate their transport MFP of 16 µm and 25 µm, respectively.

**Supplementary Fig. 15:** The total transmission coefficient as a function of scattering layer number for experimental setup in Supplementary Fig. 13b.

The experimental results show a strong scattering effect from the volumetric scatterers in chicken eggshell membranes. The membrane thickness is at least 7.3 times of scattering MFP (*l_s_*) and 3.6±0.5 times of the transport MFP (*l_t_*). The results confirm the very high opacity of the scattering media. We can also easily observe this in our Fig. 1a: the chicken eggshell membrane scatters light even more than ground glass diffusers. Unlike 3D volumetric scatterers in chicken eggshell membranes, the ground glass optical diffusers are roughened within a finite depth on one surface of thick glass substrates. The measurement of their MFP is not straightforward.

We would like to note here that the technique works best with super-strong scattering effect (no ballistic photons) within a very thin layer, where the speckle is formed, and the memory effect is large. Therefore, we can achieve very good SOSLI results for a sizeable *L/l_t_* ratio and small *l_t_* (strongly-scattering materials), while it could fail with the same (or smaller) *L/l_t_* ratio but *l_t_* is large (weakly-scattering materials). While the *L/l_t_* ratio can indicate how strong the scattering effect is, it does not present how large the memory effect region is. And SOSLI needs a sufficiently large memory effect region to cover the entire object. However, as the object for super-resolution imaging is typically small due to large magnification, it is favourable for SOSLI.

**Supplementary References**

1. Badon, A. *et al.* Smart optical coherence tomography for ultra-deep imaging through highly scattering media. *Science Advances* **2**, e1600370, (2016).

2. Stadelman, W. J. & Cotterill, O. J. *Egg science and technology*. (Avi Pub. Co., 1986).
