# Supplementary material for: Non-invasive super-resolution imaging through dynamic scattering media
Source: Nat Commun. 2021 May 25;12:3150. doi: 10.1038/s41467-021-23421-4 (PMC8149393; doi:10.1038/s41467-021-23421-4)
Supplement: Supplementary file 3 — Description of Additional Supplementary Files [file 41467_2021_23421_MOESM3_ESM.docx]

Description of Additional Supplementary Files

Title: Supplementary Movie 1

Description: Non-invasive super-resolution imaging through dynamic scattering media. Stochastic optical scattering localization imaging (SOSLI), a computational approach, reveals nanometer scaled objects hidden behind strongly scattering media.
